# Iron oxide nanoparticles promote vascular endothelial cells survival from oxidative stress by enhancement of autophagy

**DOI:** 10.1093/rb/rbz024

**Published:** 2019-06-30

**Authors:** Jimei Duan, Jiuju Du, Rongrong Jin, Wencheng Zhu, Li Liu, Li Yang, Mengye Li, Qiyong Gong, Bin Song, James M Anderson, Hua Ai

**Affiliations:** 1 National Engineering Research Center for Biomaterials, Sichuan University, Chengdu, P.R. China; 2 Shanghai Institute of Biochemistry and Cell Biology, Chinese Academy of Sciences, Shanghai, P.R. China; 3 Department of Radiology, West China Hospital, Sichuan University, Chengdu, P.R. China; 4 Department of Pathology, Case Western Reserve University, Cleveland, OH, USA; 5 Department of Macromolecular Science, Case Western Reserve University, Cleveland, OH, USA; 6 Department of Biomedical Engineering, Case Western Reserve University, Cleveland, OH, USA

**Keywords:** human umbilical vein endothelial cells, dextran-coated iron oxide nanoparticles, autophagy, oxidative stress

## Abstract

Dextran-coated superparamagnetic iron oxide nanoparticles (Dex-SPIONs) are excellent magnetic resonance imaging contrast agents for disease diagnosis and therapy. They can be delivered to target tissues mainly though vascular endothelium cells, which are major targets of oxidative stress. In cardiovascular cells, autophagy serves primarily on a pro-survival approach that protects the cells from oxidative stress even some autophagy inducers have been developed for adjuvant therapy of cardiovascular disorders. Our study demonstrated that the nanoparticles could be taken up by human umbilical vein endothelial cells (HUVECs) without causing obvious cytotoxicity but triggering autophagy. Furthermore, our results revealed that Dex-SPIONs could enhance HUVECs survival and reverse the reduction of nitric oxide secretion under the condition of H_2_O_2_ damage. However, these effects could be diminished by the autophagy inhibitor. In particular, we discovered that Dex-SPIONs evoked autophagy in HUVECs by reducing the phosphorylation of PRAS40, an upstream regulator of autophagy initiation. These results suggested that Dex-SPIONs functions as an autophagic-related antioxidant in HUVECs which may be utilized as an adjuvant therapy to cardiovascular disease associated with oxidative stress.

## Introduction

Superparamagnetic iron oxide nanoparticles (SPIONs) are excellent magnetic resonance imaging contrast agents for disease diagnosis and therapy [1, 2]. They have been widely used in clinical applications such as identifying inflamed lesions of atherosclerosis to predict future risks and prognosis [3]. After intravenous administration, SPIONs can be delivered to the inflamed lesions through blood circulation. During this process, SPIONs closely interact with vascular endothelial cells (VECs), which are also the major targets of oxidative stresses, playing a critical role in the pathophysiology of atherosclerosis as well as several other vascular diseases and disorders [4]. However, the interactions between them have rarely been studied, especially under the condition of oxidative stress.

It has been well studied that the cells can orchestrate autophagy signaling for responding to stressors induced by invaded nanoparticle [5]. This response can promote cell survival through assisting metabolism and elimination of foreign nanoparticles [6]. However, it can also accelerate cell death through excessive self-digestion with the condition of overload stressors caused by nanoparticles [7]. According to the published reports, autophagy is important for human umbilical vein endothelial cells (HUVECs) physiology functions such as VEGF and von Willebrand factor (vWF) secretion and tubule formation [8, 9]. Most importantly, autophagy predominantly acts as a critical pro-survival pathway in HUVECs against pathology conditions including oxidative stressors and high glucose via eliminating reactive oxygen species, misfolded proteins and damaged organelles [10]. However, the protective autophagy response is attenuated as the disease progresses. Some small molecular autophagy inducers, such as curcumin, resveratrol, 6-gingerol, delphinidin-3-glucoside and ampelopsin have been studied on their protective effects against oxidative stressors in damaging VECs, implying potential therapeutic potentials of these compounds on cardiovascular disorders [11–15]. Among them, curcumin and 6-gingerol could promote Beclin1 activation by inhibiting the PI3K/AKT/mTOR signaling pathway and triggering the autophagy survival response of anti-oxidative stress, so as to protect endothelial cells from oxidative stress. Resveratrol and delphinidin-3-glucoside could induce autophagy by activating the AMPK/SIRT1 signaling pathway, protecting HUVECs from oxidative damage induced by ox-LDL. Ampelopsin could activate AMPK/mTOR signaling pathway, trigger autophagy and fight against the oxidative damage of endothelial cells induced by high glucose, making it a promising treatment option for type 2 diabetes. These studies suggest that autophagy may be a potential multi-target therapeutic approach for oxidative stress-related cardiovascular diseases.

The surface coating materials used for dispersing SPIONs are key factors accounting for their biosafety. Dextran, a well-known macromolecular polysaccharide for coating of iron oxide cores, is clinically approved with excellent biocompatibility. The protective autophagy responses of dextran-coated SPIONs (Dex-SPIONs) have also been demonstrated in other cells, such as monocytes, macrophages and dendritic cells [16–18]. As a continued work, we investigated the autophagy response induced by dextran-coated SPIONs in HUVECs and studied if Dex-SPIONs could protect HUVECs from oxidative stress damage via autophagy.

## Materials and methods

### Materials

Ferrozine, neocuproine, ascorbic acid and 3-methyladenine (3-MA) were bought from Sigma-Aldrich (USA). Ammonium acetate and 30% hydrogen peroxide (H_2_O_2_) solution were purchased from Aladdin (China). Antibody against LC3 (NB100-2220) and P62 (ab56416) were purchased from Novus (USA) and Abcam (USA), respectively. PathScan^®^ Intracellular Signaling Array Kit (7323), antibody against PARP (9542T) and Beclin-1 (3495T) and goat-anti-rabbit-IgG-HRP were all obtained from Cell Signaling Technology (USA). Goat-anti-mouse-IgG-HRP was obtained from Beyotime Biotechnology (China). Antibody against β-actin (abs-118937) was bought from Absin (China).

### Preparation and characterization of Dex-SPIONs

In the study, the synthesis of Dex-SPIONs was carried out through co-precipitation method following previous literature [19]. Briefly, 3.751 g of Mw 70 000 dextran (Sangon Biotech, China) and 2.275 g ferric chloride hexahydrate (Aladdin, China) were dissolved in 30 ml of deionized water in a round bottom flask and then evacuated for 10 min. Then, 0.976 g ferrous chloride tetrahydrate (Aladdin, China) was added under an argon atmosphere, and after ultrasonic dissolution, it was transferred to a mechanically stirred three-necked flask. While being rapidly stirred, 15 ml ammonia solution was slowly dropwise added into the reaction system under the protection of argon. After the system was heated to 75°C and held for half an hour at this temperature, the reaction was stopped and the flask was naturally cooled to room temperature. The reaction product was then centrifuged at high speed for 15 min to remove large particles and dialyzed supernatant in citrate buffer. The size and zeta potential of the Dex-SPIONs were detected by a nanoparticle size and potential analyser (Zetasizer Nano ZS, Malvern, UK). The morphology of the Dex-SPIONs was characterized by scanning electron microscopy (SEM, JSM-7500F, JEOL, Japan) and transmission electron microscopy (TEM, HT-7800, Hitachi, Japan). The concentration of iron was determined by atomic absorption spectrometry (AAS, AA800, Perkin Elmer, USA).

### Cell culture

HUVECs were cultured in endothelial cell medium (ECM) containing 5% FBS, 1% ECGS, 100 U/ml penicillin and 100 μg/ml streptomycin in a humidified atmosphere of 5% CO_2_ at 37°C. HUVECs from 3 to 5 passages were used for experiments. HUVECs and ECM were both purchased from Sciencell (USA).

### Cell viability

The cell survival rate was assessed by using the CCK-8 assay kit (Dojindo, Japan). Briefly, HUVECs were first seeded in 96-well culture plates (1 × 10^4^ cells/well) and then incubated with 3-MA and Dex-SPIONs at the indicated concentrations and times. After that, the cells were oxidatively damaged by exposing to 400 μmol/L H_2_O_2_ in fresh culture medium for 4 h. Then the medium was removed carefully and replaced with fresh medium containing 10% CCK-8 for a further 2-h incubation. The absorption of mixtures at 450 nm was measured with a microplate reader (Thermo Scientific, USA). The cell viability was calculated from the optical density ratio between the experimental groups and the normal control groups. The data represent the averages of five wells per group. Each experiment was repeated at least three times individually.

### Cellular uptake of Dex-SPIONs

Intracellular iron content was determined by the colorimetric ferrozine assay [20, 21]. HUVECs were labeled with different concentrations of Dex-SPIONs (5, 10, 20, 50, 100 μg/ml) within 48-well culture plate (3 × 10^4^ cells/well) for 24 h. After washing three times with PBS, the cells were lysed by 50 mM NaOH for 2 h and then the lysis was neutralized with 10 mM HCl for 10 min. After that, the mixtures were added with iron-releasing reagent [a mixture of an equal volume of 1.4 M HCl and 4.5% (w/v) KMnO_4_ in H_2_O] and incubated at 60°C for 2 h away from light. The mixtures were then added with the iron reaction reagents (6.5 mM ferrozine, 6.5 mM neocuproine, 1 M ascorbic acid, 2.5 M ammonium acetate in water) while cooling down to the room temperature following by gently shaking for 30 min. The optical densities were read with a microplate reader at 570 nm. The iron content of each cell was calculated according to a standard protocol as previously described.

### Prussian blue staining

HUVECs were seeded in 48-well culture plates (3 × 10^4^ cells/well), and treated with Dex-SPIONs for 24 h. After that, cells were fixed with 4% paraformaldehyde for 15 min followed by washing with PBS three times. Then, the fixed cells were stained with an equal amount of 5% potassium ferrocyanide and 10% hydrochloric acid for 20 min. After washing, the nuclear fast red was counterstained for 5 min. Finally, photographs were observed with an optical microscope (Leica, Germany).

### Live/dead assay

HUVECs were seeded into 48-well plates at a density of 1.5 × 10^4^/well. After adhesion for 12 h, Dex-SPIONs at different concentrations (0, 5, 10, 20, 50, 100 μg/ml) were added to the medium for another 24-h incubation. After that, cells were incubated with 5 μg/ml propidium iodide (PI) and 1 μg/ml fluorescein diacetate (FDA) simultaneously away from light for 1–3 min. Then the cells were washed with PBS three times and analysed by fluorescent microscope.

### Immunoblotting analysis

HUVECs were seeded in 6-well culture plates (2 × 10^5^ cells/well). After adhesion, they were pretreated with 3-MA for 6 h and then incubated with Dex-SPIONs at the indicated concentrations for 24 h following by exposing to H_2_O_2_ for another 4 h in fresh medium. Then the cells were collected and lysed with cell lysis reagent. The supernatant was collected by centrifugation at 12 000 rpm for 15 min at 4°C, and dithiothreitol containing SDS buffer was added. The equivalent amount of protein from each sample was subjected to a 10% or 15% SDS-PAGE separation gel and transferred to a polyvinylidene fluoride membrane (Bio-Rad, USA). Blocking with 5% skim milk in PBST for 1 h at room temperature, then membranes were incubated overnight at 4°C with diluted primary antibody in blocking buffer. Then, the membranes were incubated with diluted secondary antibody for 1 h at room temperature. Finally, the antigen–antibody complexes were visualized using a chemiluminescence kit (Bio-Rad, USA). The bands were detected by the ChemiDoc XRS System (Bio-Rad, USA). The relative intensity of each band was normalized to the band of β-actin respectively [17].

### Transmission electron microscopy assay

HUVECs (3 × 10^6^ cells/dish) were treated with or without the nanoparticles (100 μg/ml) for 24 h. After that, cell pellets were collected and washed following by fixing with 2.5% glutaraldehyde for 2–4 h at 4°C. Then the pellets were rinsed with PBS and fixed with 1% osmic acid at room temperature for 2 h. The samples were then continuously dehydrated with 50%, 70%, 80%, 90%, 100% alcohol and 100% acetone and embedded in epoxy resin overnight to prepare ultrathin sections. Finally, the ultrathin sections were stained for 15 min with lead citrate and 2% uranyl acetate, respectively, and photographed using TEM (HT-7800, Hitachi, Japan).

### Measurement of nitric oxide levels in cell

Nitric oxide (NO) test kit was purchased from Nanjing Jiancheng Institute (A012). HUVECs were seeded in 48-well culture plates at a density of 3 × 10^4^ cells/well and treated as described in the above sections. Then the nitric oxide levels in cell supernatants were detected according to the manufacturer’s instructions.

### Apoptotic intracellular signaling arrays

Cells were incubated with the nanoparticles (100 μg/ml) for 24 h and then treated with or without 400 μM√ H_2_O_2_ for another 4 h. The apoptotic intracellular signals were measured by the PathScan^®^ Intracellular Signaling Array Kit according to the manufacturer’s instructions. First, the cell lysates were added to the nitrocellulose membrane window of the antibody slide and cover with sealing tape. After the overnight incubation at 4°C with continuous and gentle shaking, the slide was thoroughly washed at room temperature for dissociation of non-specific absorption. Then the Detection Antibody Cocktail was added and incubated with the membrane at room temperature for 1 h. Next, four times of washes were performed and each well was incubated for 30 min with HRP-linked Streptavidin. Afterward, the slide was washed and covered with the LumiGLO^®^/Peroxide reagent. Finally, the images of the slide were captured by using a ChemiDoc Touch System (Bio-Rad, USA). The relative intensity of each spot was normalized to positive control spots.

### Statistical analysis

Data are shown as mean ± standard deviation of at least three independent experiments. A two-tailed *t*-test was used for statistical comparison between groups. A *P* < 0.05 indicates that the difference was statistically significant.

## Results and discussion

### Characterization of Dex-SPIONs

**Figure 1:**
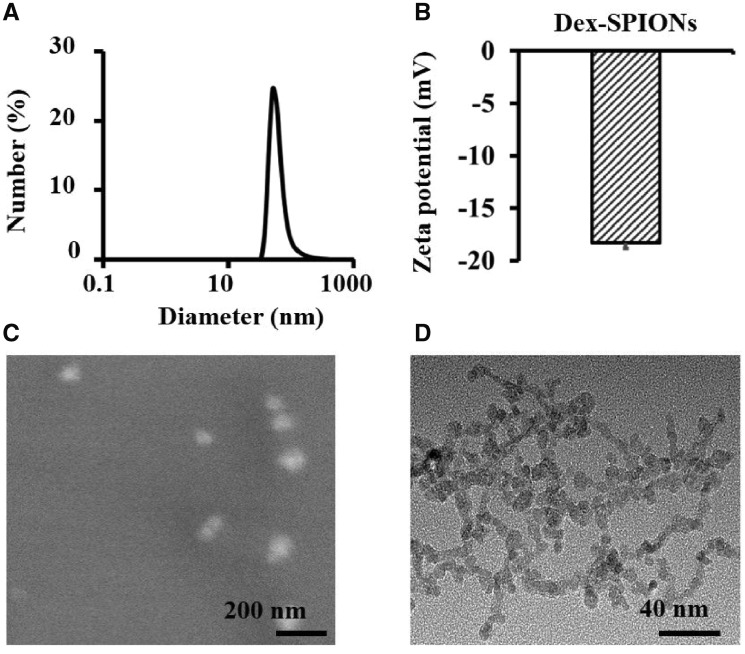
Physical characterization of Dex-SPIONs. (**A**) DLS of Dex-SPIONs in water, (**B**) zeta potential of Dex-SPIONs, (**C**) SEM of Dex-SPIONs and (**D**) TEM of Dex-SPIONs

Dex-SPIONs were fabricated by the aqueous-phase co-precipitation method as described above. The size and zeta potential of Dex-SPIONs were measured by dynamic light scattering (DLS). The morphology of Dex-SPIONs was characterized by SEM and TEM. As shown in Fig. 1A,√ the mean hydrodynamic size of Dex-SPIONs was about 65.3 nm, and the surface charge of Dex-SPIONs was approximate ‒18.3 mV (Fig. 1B). The negative charge of nanoparticles was probably due to the lower acidity of organic alcohol groups and hydrated iron oxide cores [22]. SEM analysis showed that the morphology of the Dex-SPIONs featured spherical particles with uniform size and good dispersion in water without obvious agglomeration (Fig. 1C). The iron oxide core in dextran had a uniform appearance and a diameter of about 5–10 nm (Fig. 1D), smaller than the hydrodynamic diameter shown in Fig. 1A.

### Intracellular iron content and cell viability evaluation

**Figure 2:**
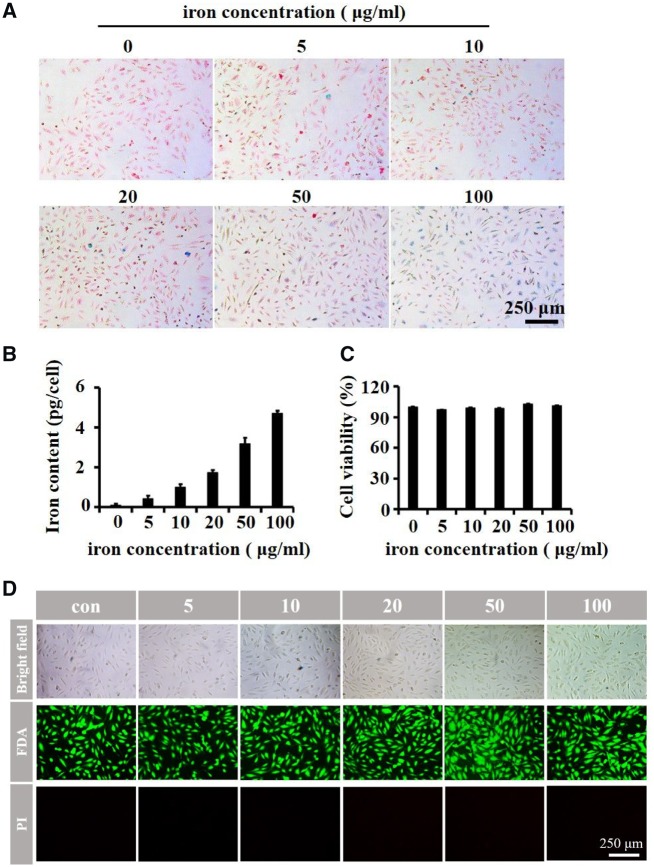
The cell iron content and viability of HUVECs after labeling with Dex-SPIONs for 24 h. (**A**) Prussian blue staining of HUVECs after incubating with Dex-SPIONs at the indicated concentrations for 24 h. (**B**) Measurement of intracellular iron content after incubation for 24 h with the indicated concentrations of Dex-SPIONs in HUVECs. (**C**) The cells viability after incubation with Dex-SPIONs for 24 h at the indicated concentrations were determined using CCK-8. (**D**) Live/dead staining of HUVECs after incubating with Dex-SPIONs at the indicated concentrations for 24 h

To our best knowledge, there are four different dextran-coated SPIONs have been clinically approved, namely Feridex, Resovist, Feraheme and Abdoscan. After administration, they are mainly trapped by macrophages in the reticuloendothelial system (RES) organs. During delivery, Dex-SPIONs are frequently in contact with endothelial cells of blood vessels. However, the interactions have rarely been studied. So we first conducted experiments to investigate the uptake behavior of Dex-SPIONs by HUVECs. As shown in Fig. 2A, HUVECs can efficiently internalize Dex-SPIONs when the iron concentration was higher than 50 μg/ml, illustrated by blue areas for iron staining and red areas for nucleus staining.

In addition, it was observed that the iron content in HUVECs increased in a dose-dependent manner as the concentration of iron ions increased (Fig. 2B). After a 24-h incubation with 100 μg/ml Dex-SPIONs, the internalized iron content per cell was approximately 5 pg, compared to about 5 pg Fe/cell in human peripheral blood monocytes, 4 pg Fe/cell in mesenchymal stem cells and 48 pg Fe/cell in bone-marrow derived macrophages in the same condition, exhibiting a relative strong phagocytic ability [16, 17, 23]. As previously reported, lauric acid-coated iron oxide nanoparticles reduced endothelial cell viability at the iron concentration exceed 50 μg/ml for 24 h treatment [24]. Therefore, the cell viability of HUVECs after treated with √Dex-SPIONs was evaluated subsequently. As shown in Fig. 2C, dextran-coated iron oxide nanoparticles were secure toward HUVECs’ viability, indicating dextran might be safer than lauric acid as coating materials. In addition to the CCK-8 experiment, cell live/dead staining also was performed to analyse the effect of Dex-SPION on cell viability. As shown in Fig. 2D, the cell morphology of nanoparticles treated groups does not show obvious change comparing to the control group and PI positive cells, representing the dead ones, are not observed in the fluorescence images of HUVECs in all groups analysed by live/dead assay. These results further indicated that dextran-coated Dex-SPIONs exhibit excellent biocompatibility to HUVECs.

### Dex-SPIONs promoted autophagy in HUVECs

**Figure 3:**
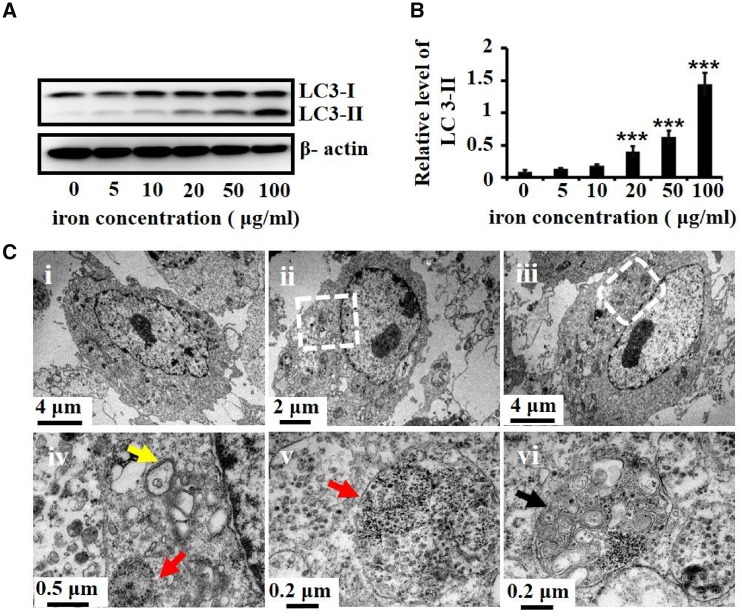
Autophagy response induced by Dex-SPIONs in HUVECs. (**A**) Immunoblot analysis of endogenous LC3 proteins after incubation with Dex-SPIONs at the indicated concentrations for 24 h. β-Actin served as loading control. (**B**) The levels of LC3-II were quantitatively analysed by density analysis. Values were represented as the mean ±* *SD, *n *= 3. ***P < 0.001 versus control. (**C**) TEM analysis of autophagy flux in HUVECs; untreated HUVECs (i) and Dex-SPIONs (100 μg/ml, 24 h) treated HUVECs (ii–vi); zoom-in of ii (iv) and zoom-in of iii (v and vi); intracellular accumulation of Dex-SPIONs in endosomes (red arrows), formation of early autophagic vacuole (yellow arrow) and autolysosome (black arrow)

As described above, HUVECs can efficiently internalize Dex-SPIONs. Previous studies have shown that cells can orchestrate autophagy signaling for responding to stressors induced by foreign nanoparticles [25]. We then conducted experiments to detect whether HUVECs would induce autophagy response toward Dex-SPIONs treatment. First, we examined the conversion of LC3 proteins in the cells after incubating with Dex-SPIONs at various concentrations, which will be transformed to LC3-II by the phosphatidylethanolamine conjugation for autophagosome formations. As shown in Fig. 3A and B, Dex-SPIONs could visibly cause LC3-II accumulation in a dose-dependent manner in HUVECs. In addition, TEM, a gold standard of autophagy detection, was applied to observe the formation of autophagic vesicles in HUVECs. As shown in Fig. 3C, HUVECs treated with Dex-SPIONs presented endosomes with highly electron-dense nanoparticles (Fig. 3C, v and iv, red arrows) and autolysosomes with electron-dense nanoparticles, organelle and cell debris (Fig. 3C, vi, black arrow). Furthermore, double-membraned autophagic vacuoles with some cell fragments were also found in the cells. (Fig. 3C, iv, yellow arrow). These results indicated that complete autophagic flux in HUVECs was enhanced by the response to Dex-SPIONs.

### Dex-SPIONs play a protective role on oxidative stress damaged HUVECs

**Figure 4:**
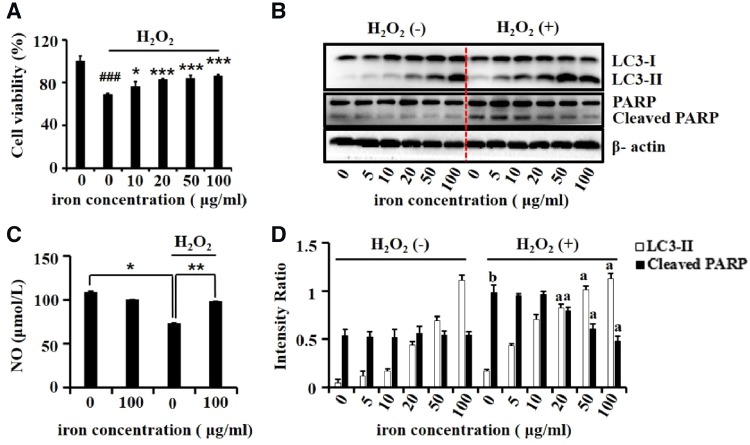
The protective effects of Dex-SPIONs on H_2_O_2_-induced damage in HUVECs. (**A**) Rescue effects of Dex-SPIONs on the viability of HUVECs exposure to H_2_O_2_. HUVECs were incubated with Dex-SPIONs at the indicated concentrations for 24 h following by exposing to H_2_O_2_ (400 μmol/L) for another 4 h. Then the cell viability was determined by CCK-8 assay. Values are expressed as the mean ± SD, *n* = 5. ^###^*P* < 0.001 versus control, *P < 0.05, ***P < 0.001 versus H_2_O_2_ alone group. (**B**) Immunoblot analysis of LC3 and PARP in Dex-SPIONs treated HUVECs at the indicated dosages. β-Actin was served as loading control. (**C**) Effects of Dex-SPIONs on the NO generation of HUVECs treated with H_2_O_2_. *P < 0.05, **P < 0.01. (**D**) The levels of LC3-II and cleaved PARP were quantitatively analysed by density analysis. Value was shown as mean ± SD, *n* = 3. ^a^*P* < 0.001 versus H_2_O_2_ alone group; ^b^*P* < 0.001 versus control

As Dex-SPIONs could induce autophagy responses in HUVECs and such responses can protect HUVECs from oxidative stress damages [26], we then conducted experiments to evaluate whether Dex-SPIONs could play a protective role on oxidative stress damaged HUVECs. The CCK-8 analysis was used to determine cell viability (Fig. 4A), showing that Dex-SPIONs could partially restore the cell survival from H_2_O_2_ induced repression in a dose-dependent mode. Not surprisingly, Dex-SPIONs could decrease the induction of H_2_O_2_ on cleavage of PARP, which served as an apoptotic indicator [27] (Fig. 4B and D). Interestingly, Dex-SPIONs triggered more LC3-II transformation in H_2_O_2_ treated group than untreated ones whereas H_2_O_2_ itself cannot induce LC3-II transformation (Fig. 4B and D), probably indicating that the nanoparticles facilitate the activation of autophagy flux in HUVECs when damaged by H_2_O_2_. NO is a gas signal molecule produced by HUVECs after activation of endothelial nitric oxide synthase (eNOS) [28]. It can expand blood vessels, inhibit the aggregation and adhesion of platelets as well as the proliferation and migration of vascular smooth muscle cells [29]. Therefore, NO is one of the key factors to maintain the homeostasis of HUVECs and vascular system. As illustrated in Fig. 4C, Dex-SPIONs could reverse the reduced production of NO caused by H_2_O_2_ damage, indicating a protective role of Dex-SPIONs on injured HUVECs.

### Dex-SPIONs induced protective autophagy in HUVECs

**Figure 5:**
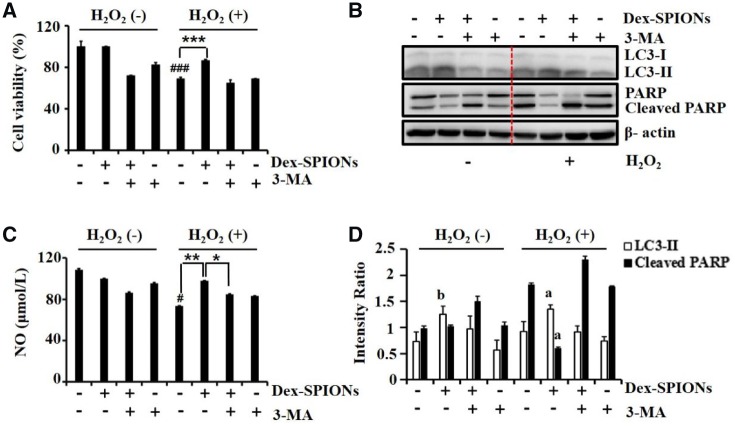
The effects of autophagy inhibitor 3-MA on protective autophagy induced by Dex-SPIONs in H_2_O_2_ treated HUVECs. (**A**) Cell viability of HUVECs after pretreated with 3-MA (10 mM) for 6 h and incubated with Dex-SPIONs (100 μg/ml) for 24 h then exposed to H_2_O_2_ (400 μM) for 4 h. Data were shown as mean ± SD, *n* = 5. ^###^*P* < 0.001 versus control, ***P < 0.001 versus H_2_O_2_ alone group. (**B**) Immunoblot analysis of LC3-II and PARP in HUVECs which were treated as same as the processing condition of (A). β-Actin was served as loading control. (**C**) Effects of Dex-SPIONs on the NO generation of HUVECs which were treated as same as the processing condition of (A). ^#^*P* < 0.05 versus control, **P < 0.01, *P < 0.05. (**D**) The levels of LC3-II and cleaved PARP were quantitatively analysed by density analysis. Data were presented as means ± SD (*n* = 3); ^a^*P* < 0.001 versus H_2_O_2_ alone group; ^b^*P* < 0.001 versus control

To verify whether the protective effect of Dex-SPIONs in H_2_O_2_ damaged HUVECs was relying on their autophagy response, we used 3-MA, a classic autophagy inhibitor [22]. We treated HUVECs with Dex-SPIONs for 24 h, of which some cells were pretreated with 10 mM 3-MA for 6 h, and then exposed them to H_2_O_2_ for 4 h. Compared with the injured group, the cell viability of Dex-SPIONs treated group showed a significant recovery from oxidative stress. However, the recovery was observably inhibited by 3-MA treatment (Fig. 5A). Then, the levels of LC3-II and PARP were examined in the same conditions. As shown in Fig. 5B, 3-MA reduced the accumulation of LC3-II in Dex-SPIONs treated HUVECs with or without H_2_O_2_ damage. Simultaneously, it promoted the induction of cleaved PARP which was decreased by Dex-SPIONs while treated with H_2_O_2_. In addition, discernible activation of cleaved PARP was observed in the group incubated with 3-MA and H_2_O_2_ successively, sustaining the idea that basic autophagy is critical for HUVECs survival in the condition of oxidative damage. Besides, the release of NO was also detected after the same treatment. As shown in Fig. 5C, 3-MA inhibited the recovery of NO caused by Dex-SPIONs in H_2_O_2_ damaged HUVECs. These data indicated that 3-MA could suppress the rescue effect of Dex-SPIONs in H_2_O_2_ damaged HUVECs, including their survival and NO release. We could conclude that the protective effect of Dex-SPIONs in H_2_O_2_ damaged HUVECs largely relies on their autophagy response.

### Intracellular signaling pathways of Dex-SPIONs anti-apoptosis in H_2_O_2_ damaged HUVECs

**Figure 6:**
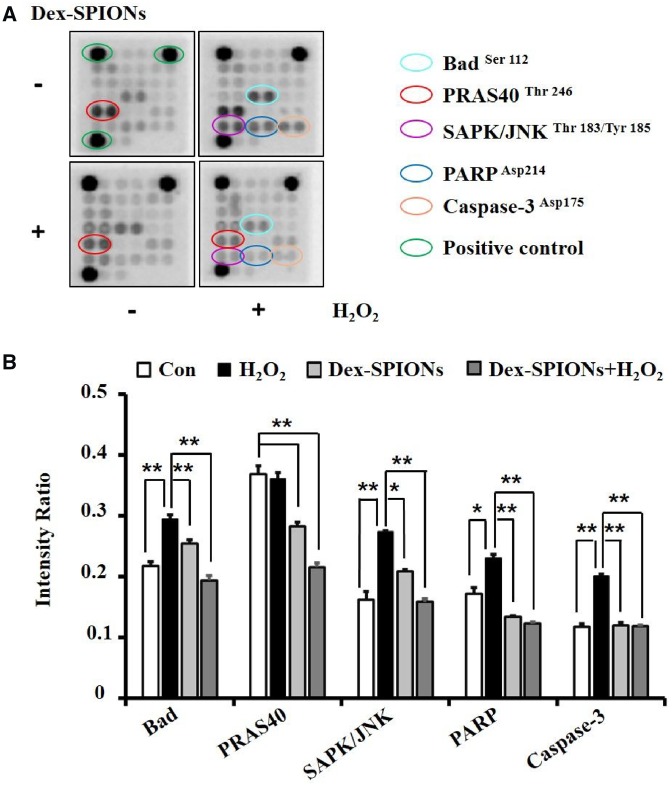
The effects of Dex-SPIONs and H_2_O_2_ on phosphorylation or cleavage of apoptotic signaling proteins. (**A**) The chemiluminescent images obtained by using the PathScan^®^ RTK signaling antibody array kit revealing various phosphorylated or cleaved signaling nodes in HUVECs treated with Dex-SPIONs (100 μg/ml, 24 h) and H_2_O_2_ (400 μM, 4 h) alone or collectively. (**B**) The levels of bad, SAPK/JNK, PARP and caspase 3 were quantitatively analysed by density analysis. Positive controls were acted as the loading control. **P < 0.01, *P < 0.05

**Scheme 1:**
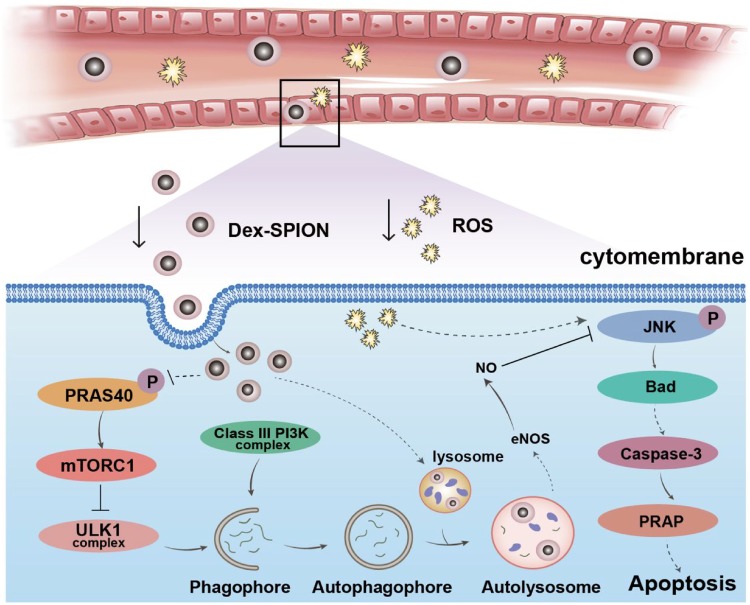
A schematic model of Dex-SPIONs protect HUVECs from H2O2 damage via inducing autophagy response. After intravenous injection, Dex-SPIONs can be internalized by HUVECs, resulting in the induction of autophagy flux by inhibiting the phosphorylation of PRAS40, an upstream autophagy blocker of autophagy through activating mTORC1. Then, NO generation is increased through upregulation of autophagy, probably due to the increased expression of eNOS. Eventually, the upregulated NO inhibits the phosphorylation of JNK and its downstream apoptotic pathway, leading to restoration of cell viability from oxidative damage

To explore the mechanism of Dex-SPIONs anti-apoptosis in H_2_O_2_ damaged HUVECs, the PathScan^®^ Intracellular Signaling Array Kit was used to simultaneously detect the expression of 18 signaling molecules that are closely related to autophagy and apoptosis, of which the active form was phosphorylated or cleaved. The representative array membranes were shown in Fig. 6A after reacting with cell lysates of different groups and signals exhibited activation or inactivation were highlighted. Dot intensity quantified analysis showed that the Dex-SPIONs could downregulate the phosphorylation of pro-apoptotic protein Bad and SAPK/JNK which were increased by H_2_O_2_ incubation in HUVECs. Meanwhile, they can diminish the cleavage of apoptotic protein Caspase-3 and PARP induced by H_2_O_2_. In addition, the phosphorylation level of PRAS40 was reduced in Dex-SPIONs treated groups. It was reported that PRAS40 could be a substrate of protein kinase B (PKB/Akt) and a specific binding protein of mTOR complex 1 (mTORC1) [30]. Phosphorylation of PRAS40 at Thr246 can relieve it from inhibition of mTORC1, inhibiting autophagy by the inhibitory effect on the ULK1 complex [31]. Therefore, it was speculated that Dex-SPIONs might promote autophagy by reducing the phosphorylation of PRAS40, consistent with the reported results that iron oxide nanoparticles can induce autophagy by inhibiting mTOR pathway [25]. It has also been reported that autophagy contributes to an increased eNOS expression [32] to generate NO for signal transduction by inhibiting the phosphorylation of SAPK/JNK, ultimately resulting in attenuated apoptosis [33]. In conclusion, Dex-SPIONs could protect HUVECs from apoptosis through inhibition of SAPK/JNK phosphorylation and its downstream apoptotic signals, due to the NO generation induced by the corresponding autophagy response (Scheme 1).

## Conclusions

Autophagy plays an important role in physiological functions and pathological processes of the organisms. In this study, we found that Dex-SPIONs could enhance VEC survival and reverse the reduction of NO secretion under the condition of H_2_O_2_ damage. However, these effects could be diminished by autophagy inhibitor, indicating that the protective role of Dex-SPIONs in H_2_O_2_ damaged HUVECs was dependent on their autophagy response. In particular, we discovered that Dex-SPIONs triggered autophagy in HUVECs by reducing the phosphorylation of PRAS40 which served as an agonist of mTORC1, a critical upstream blocker of autophagy initiation. The elevated autophagy response redounded the generation of NO, leading to the blockage of JNK phosphorylation and its downstream apoptotic signals. Our study results suggested that Dex-SPIONs may be a promising candidate for adjuvant therapy to lower the oxidative damage in cardiovascular diseases and disorders.

## Funding

This work was supported by the Innovative Research Groups of the National Natural Science Foundation of China (no. 81621003), National Key Basic Research Program of China (no. 2013CB933903), Sichuan Science and Technology Program (no. 2019JDRC0103) and China Postdoctoral Science Foundation Funded Project (no. 2015M572475).

*Conflict of interest statement*. None declared.
